# Modeling *GATAD1*-Associated Dilated Cardiomyopathy in Adult Zebrafish

**DOI:** 10.3390/jcdd3010006

**Published:** 2016-01-26

**Authors:** Jingchun Yang, Sahrish Shah, Timothy M. Olson, Xiaolei Xu

**Affiliations:** 1 Department of Biochemistry and Molecular Biology, Mayo Clinic College of Medicine, 200 First St. SW Rochester, MN 55905, USA; yang.jingchun@mayo.edu (J.Y.); shah.sahrish@mayo.edu (S.S.); 2 Department of Internal Medicine, Division of Cardiovascular Diseases, Mayo Clinic College of Medicine, 200 First St. SW Rochester, MN 55905, USA; Olson.Timothy@mayo.edu; 3 Department of Pediatrics and Adolescent Medicine, Division of Pediatric Cardiology, Mayo Clinic College of Medicine, 200 First St. SW Rochester, MN 55905, USA

**Keywords:** adult zebrafish, cardiomyopathy, *gatad1*, TALEN, transgenic fish

## Abstract

Animal models have played a critical role in validating human dilated cardiomyopathy (DCM) genes, particularly those that implicate novel mechanisms for heart failure. However, the disease phenotype may be delayed due to age-dependent penetrance. For this reason, we generated an adult zebrafish model, which is a simpler vertebrate model with higher throughput than rodents. Specifically, we studied the zebrafish homologue of *GATAD1*, a recently identified gene for adult-onset autosomal recessive DCM. We showed cardiac expression of *gatad1* transcripts, by whole mount *in situ* hybridization in zebrafish embryos, and demonstrated nuclear and sarcomeric I-band subcellular localization of Gatad1 protein in cardiomyocytes, by injecting a Tol2 plasmid encoding fluorescently-tagged Gatad1. We next generated *gatad1* knock-out fish lines by TALEN technology and a transgenic fish line that expresses the human DCM *GATAD1*-S102P mutation in cardiomyocytes. Under stress conditions, longitudinal studies uncovered heart failure (HF)-like phenotypes in stable KO mutants and a tendency toward HF phenotypes in transgenic lines. Based on these efforts of studying a gene-based inherited cardiomyopathy model, we discuss the strengths and bottlenecks of adult zebrafish as a new vertebrate model for assessing candidate cardiomyopathy genes.

## 1. Introduction

Cardiomyopathy refers to cardiac disease that is associated with structural changes of the myocardium upon extrinsic stresses such as ischemia and hypertension, and intrinsic stresses such as genetic mutations, many of which ultimately lead to heart failure [1]. Depending on the nature of the biomechanical stressors, various remodeling phenotypes can occur, including hypertrophic cardiomyopathy (HCM), dilated cardiomyopathy (DCM), and restrictive cardiomyopathy (RCM). Human genetic studies have identified mutated genes responsible for 50%–70% of HCM, 30%–50% of DCM, and a small fraction of RCM [2,3], which create opportunities to pinpoint disrupted signaling pathways and seek therapeutic strategies. Beyond hypothesis-based candidate gene approaches, the development of genomic technologies including genome-wide association studies, family-based locus mapping, whole-exome sequencing, and chromosomal microarray have facilitated discovery of novel, unsuspected susceptibility genes [4]. However, these strategies may yield multiple plausible candidate genes in individual families and/or require a higher burden of proof to establish causality. An efficient animal model with high throughput is needed to assess these candidate genes and to expedite the gene discovery process.

The mouse is currently the preferred animal to model cardiomyopathy, but the associated high costs and low throughput limit its application for discovering and validating new cardiomyopathy genes. As reviewed by other papers in this special issue, Drosophila is a highly efficient invertebrate model that has contributed significantly to our understanding of cardiogenesis and adult heart diseases. However, the tubular structure of a fly heart and the fact that 39% of human genes are missing in fly genome impose significant limitations on assessing candidate genes for cardiomyopathy [5,6]. In contrast, the zebrafish heart can be thought of as a simplified human heart, consisting of a single atrium, ventricle, and outflow tract [7]. Major cardiac structures including the myocardium, endocardium, epicardium and cardiac valves are conserved in this vertebrate model. Besides structural conservation, conservation at the molecular level has been supported by a comprehensive scan of the zebrafish genome to identify homologues of known human DCM genes. Ninety-six percent of known human DCM genes (49 of 51) have a corresponding zebrafish homologue, of which 30 have a single homologue [8]. For 14 out of the remaining 19 genes with multiple homologues, a single zebrafish homologue has been prioritized from other homologues by scoring its cardiac abundance and enrichment based on RNAseq analysis [8]. Together, these data lay a foundation for generating genetic cardiomyopathy mutants in adult zebrafish.

The use of zebrafish as an animal model to study cardiomyopathy was initially reported in 2002, when Xu *et al.* identified a *titin* mutant and *Sehnert* *et al.* cloned a *tnnt2* mutant, representing two embryonic zebrafish forms of cardiomyopathy [9,10]. Because of efficient technologies that can be applied during zebrafish embryogenesis, such as morpholinos to knock-down gene expression and chemical screening platforms to search for phenotypic modification, zebrafish have been successfully used to validate candidate genes identified by human genetic studies [11,12], discover new cardiomyopathy genes [13], and seek potential therapeutic strategies [14,15]. However, fish embryos have intrinsic limitations. First, the short developmental time window prevents fish embryos from faithfully recapitulating the pathogenic process of cardiomyopathy, which typically involves age-dependent penetrance and gradual progression to overt heart failure in adulthood. Second, the inheritance patterns of cardiomyopathy can be diverse, including autosomal dominant, autosomal recessive (homozygous and compound heterogeneous), and X-linked recessive, and difficult to recapitulate by assessing embryonic phenotypes upon near-null gene depletion. It is estimated that only 5%–10% of genes exhibit embryonic-lethal phenotypes upon depletion [16]. Third, there are significant concerns about the specificity of the morpholino technology, which might incur pathological changes different from stable mutants [17,18]. Having recognized the shortcomings of the fish embryo, Sun *et al.* started to explore whether cardiac remodeling exists in an adult zebrafish heart [19]. Detailed characterization of *tr265*, a mutant with a defective *band 3* gene [20], showed that high-output cardiac stress from chronic anemia causes significant enlargement of the ventricular chamber. Hallmarks of cardiomyopathy, including reduced ejection fraction, muscular disarray, and fetal gene reactivation, were evident [19,21]. At the cellular level, cardiomyocyte (CM) hypertrophy and activated CM proliferation have been detected. Ding *et al.* reported that injection of doxorubicin (DOX), a widely used anticancer drug that induces cardiomyopathy in humans and rodent models [22,23], likewise induces ventricular enlargement in adult zebrafish and is associated with the hallmarks of cardiomyopathy [24]. Strong activation of CM apoptosis and unchanged CM proliferation was noted, indicating that the pathogenic mechanisms differed between the DOX and anemia models. The successful generation of two acquired-cardiomyopathy models with different pathogenesis demonstrated that the simple zebrafish heart is likely complex enough to model different types of cardiomyopathy. Consistent with this concept, a transgenic fish line that expresses a human 2057del2 mutation in the gene encoding Plakoglobin recapitulates perspectives of arrythmogenic cardiomyopathy [25]. These reports justify the present work to further explore genome editing technology to model cardiomyopathy in adult zebrafish.

Historically, most zebrafish stable mutants have been generated by either large scale mutagenesis screening or TILLING technology [26,27,28]. Both strategies depend on generating random mutations in the whole genome, which is only feasible by collaborative efforts in large research centers. The emergence of genome editing technology, as represented by transcription activator-like effector nuclease (TALEN) and/or CRISPR/Cas9-based technologies [29,30,31], have made it possible to genetically edit specific genes in an individual laboratory. In addition to nonsense truncation mutations, missense mutations can also be efficiently generated by using TALEN-based knock-in (KI) technology and/or transgenic technology in zebrafish [32,33,34].

GATA zinc finger domain containing 1 (Gatad1), also called (ocular development-associated gene (ODAG), was first cloned from mouse, in which Gatad1 is ubiquitously expressed in all tissues with the highest levels in testis and lung. During development, mouse Gatad1 has a dynamic expression in the eye and overexpression of Gatad1 impairs retinal development [35,36]. Through homozygosity mapping and exome sequencing, Theis *et al.* identified a GATAD1 mutation from patient cohorts with autosomal recessive dilated cardiomyopathy [37]. Interestingly, GATAD1 is highly overexpressed in female idiopathic dilated cardiomyopathy (IDCM) patients comparing to male patients, suggesting GATAD1 might contribute to gender difference in DCM patients [38].

Given that genetic models of cardiomyopathy in adult zebrafish have scarcely been reported, we leveraged genome editing technology to systematically generate stable mutants for known cardiomyopathy genes. Based on our experience with the Golden Gate custom array platform [39], TALEN constructs for five to 10 genes can be generated in two weeks, and stable knock-out (KO) zebrafish mutants can be obtained for most of them in about three months (data not published) [40]. Here, we report our study of GATAD1, a recently established gene for human DCM. In a family with adult-onset DCM, a homozygous recessive missense mutation—S102P—was discovered by locus mapping and whole exome sequencing [37]. We determined the tissue distribution of the *gatad1* transcript and the subcellular localization of the zebrafish Gatad1 protein. We then generated stable *gatad1* knock-out fish by TALEN technology and transgenic fish lines that overexpressed human GATAD1-S102P. Heart failure-like phenotypes were noted in these genetically manipulated fish after stress. Based on these data, we review adult zebrafish as an emerging vertebrate model for assessing candidate genes for cardiomyopathy and discuss the bottlenecks of this model that need to be overcome.

## 2. Experimental Section

### 2.1. Zebrafish Lines and Maintenance

Wild type WIK fish were used for this study. The IACUC approval number is A32413.

### 2.2. Bioinformatics Analysis

DSgene was used to obtain the alignments and phylogenetic tree for gatad1 sequences from different species. The Gatad1 protein sequences from the following species have been used for alignment: Homo sapiens (NM_021167), Rattus norvegicus (XP_575359); Mus musculus (NP_080309), Xenopus laevis (NM_001087134), Danio rerio (NM_001040364), Drosophila melanogaster (NP_569851), Oncorhynchus mykiss (CDQ77377), Oreochromis niloticus (XP_003444124), Takifugu rubripes (XP_003969420), Oryzias latipes (XP_004074272), Astyanax mexicanus (XP_007245785), Pundamilia nyererei (XP_005728478), Xiphophorus maculatus (XP_005797418), Haplochromis burtoni (XP_005917525), Neolamprologus brichardi (XP_006787902).

### 2.3. Whole-Mount in Situ Hybridization

Whole mount *in situ* hybridization was performed as previously described [41]. The following primer pair was used for generating *gatad1* riboprobes: forward primer 5′-TATCATGGTAAAAC ATACGGGACC-3′ and reverse primer 5′-GGTAATACGACTCACTATAGGCATGGACTCTGATGT GATGATCGTTG-3′.

### 2.4. Immunostaining

*myl7: Gatad1-GFP* construct was injected into 1-cell staged fish embryo. GFP signal starts to express at 2 dpf, when the heart was dissected from fish embryo and stained as described before [42]. The primary antibodies are α-actinin (Sigma, 1:200, St. Louis, MO, USA), egfp (Invitrogen, 1:200, Grand Island, NY, USA), myomesin (DSHB, 1:20, Iowa City, IA, USA), and then Alexa-conjugated secondary antibodies were applied (Invitrogen, 1:1000). Alexa conjugated phalloidin (Invitrogen, 1:200) was used to stain actin filament.

Frozen sections from adult fish hearts were stained with Alexa conjugated phalloidin (Invitrogen, 1:200) as described before [19].

### 2.5. Real-Time Reverse Transcription PCR

Different tissues including brain, heart, intestine, liver, muscle and spleen were dissected from adult zebrafish, and homogenized in Trizol using the Advanced Blender system with RNase-free 0.5 mm steel beads (Advanced Blending Solution, Wallace, MI, USA). Total RNAs were extracted from these tissues using the RNeasy mini kit (Qiagen, Frederick, MD, USA), and RNAs were used as templates for reverse transcription using the SuperScript^®^ III Reverse Transcription kit (Invitrogen, Grand Island, NY, USA). Real-time PCR was performed with the LightCycler^®^ 480 System (Roche, Indianapolis, IN, USA) using the following primers. For *gatad1*, forward primer: 5′-CAGGA AATACACAGACGCTC-3′ and reverse primer: 5′-AGTACTGATCCTGGACGAATC-3′; For *18s*, forward primer: 5′-TCGCTAGTTGGCATCGTTTATG-3′ and reverse primer: 5′-CGGAGGTTCG AAGACGATCA-3′; for *gapdh*, forward primer: 5′-CCACCCATGGAAAGTACAAG-3′ and reverse primer: 5′-CTCTCTTTGCACCACCCTTA-3′; for *nppb*, *vmhc* and *vmhcl*, the primer sequences have been described before [8].

### 2.6. Generation of TALEN Mutants

To generate TALEN knock-out zebrafish, the 2nd exon of *gatad1* (ENSDARG00000027612) was selected as the targeted site. This exon corresponds to the human exon that has the S102P mutation. The TALEN RNA was generated using a Golden Gate kit (Addgene, Cambridge, MA, USA) according to the instructions from the manufacturer. Briefly, the targeted exon was amplified and sequenced to ensure that no genetic variants were present in the targeted region. The TALEN RNA binding site was predicted using the Zifit website [43]. Based on the binding sequences, final constructs that encoded the desired TALEN RNAs were generated through recombining several plasmids provided in the Golden Gate kit. Using the linearized final construct as a template, the TALEN RNA was synthesized using an mMESSAGE mMachine T3 kit (Ambion, Pittsburgh, PA, USA). For the allele with the 4 nucleotides deletion, the binding sequence for the TALEN left arm is 5′-TGCAGTCCAAACAGGAAA-3′, and the binding sequence for the right arm is 5′-AGGTTAAGAAGCACTAA-3′. The targeted site contains a recognition site for the HgaI restriction enzyme. For the allele with a 13 nucleotide deletion, the binding sequence for the TALEN left arm is 5′-TTAAGAAGCACTAAGTAT-3′, for the right arm is 5′-AAAAAAAAGTCTCCACCA-3′, and the central targeted site contains a cut site for MspI. The primers for genotyping of both alleles are: forward: 5′-TTCTTTGCAGTCCAAACAGG-3′ and reverse: 5′-AAAGAGGGTCCAACCAGGTACTAG-3′.

To generate stable TALEN mutant fish, TALEN RNAs were injected at 10 ng/µL into single-cell staged embryos. Founder fish containing deletions were identified by genotyping with restriction enzymes, followed by sequencing to determine the precise molecular nature of the genomic change. Founder fish with the desired mutation were outcrossed to produce an F1 generation, which was then used for breeding to generate homozygous mutants. Homozygous mutant fish were identified by restriction fragment length polymorphism based genotyping.

### 2.7. Generation of Transgenic Fish Lines

To generate the Tol2 (*myl7:gatad1-gfp*) construct, zebrafish *gatad1* open reading frame sequence (NM_001040364) was cloned into a gateway middle entry clone pENTR1A (Invitrogen) before it was recombined with the Tol2 destination vector, p5E-myl7 vector, and the p3E-gfp vector [34]. To generate Tol2 (*myl7:GATAD1-HA IRES-GFP*) and Tol2 (*myl7:GATAD1-HA-S102P IRES-GFP*) constructs, human *GATAD1* (NM_021167) was cloned into the pENTR1A vector using the following primer pair: forward: 5′-TTAGGATCCGCCACCATGCCGCTGGGCCTGAAGCCCACC-3′ and reverse: 5′-GGTGAATTCTCAAGCGTAATCCGGAACATCGTATGGGTACATCAAATGGTTGG CAACTGATTCC-3′. An HA tag was included at the carboxyl terminal of *GATAD1*. Next, the S102P mutation was introduced into the wild type *GATAD1* by two-step PCR using the following primers: forward: 5′-AAATACAAACCTGCTCCGGCTGCTGAAAAGAAAGTC-3′, reverse: 5′-AGCCGGAG CAGGTTTGTATTTAGTGTTTCTGAGCCG-3′, and the primer pairs used for cloning wild type *GATAD1*. Finally, the pENTR1A-based constructs containing either wild type *GATAD1* or mutant *GATAD1* were recombined into the Tol2 destination expression vector, together with the p5E-*myl7* and p3E-*IERS-GFP* vectors using the Gateway system (Invitrogen). To generate stable transgenic fish, the Tol2 expression constructs together with transposase RNA were injected into fish embryos at the 1-cell stage. The injected fish were raised to the adult stage and the positive fish were selected based on the GFP reporter.

### 2.8. Stressing Fish with a High-Cholesterol Diet

A 4% cholesterol diet was made as previously descripted [44,45]. Briefly, 4 g cholesterol (Sigma, St. Louis, MO, USA) was dissolved in 200 mL ether before being mixed with 100 g of artificial artemia. The mixture was put close to the ventilation opening in the hood to evaporate until completely dry. The fish were fed with this high cholesterol diet twice a day beginning at 5 weeks of age.

### 2.9. Stressing Zebrafish Embryos with Ethanol

To impose a cardiomyopathy-inducing stressor in zebrafish, we treated the fish with ethanol during embryogenesis [46]. Briefly, the fish embryos were immersed in 0.3% ethanol from 2 hpf to 48 hpf. The embryos that did not show a phenotype after ethanol treatment were raised up to the adult stage in normal fish water.

### 2.10. Swimming Capacity Test

The fish were starved for 24 h before being placed in a swim tunnel respirometer (Mini Swim-170, Loligo Systems, Tjele, Denmark). After being acclimated to the respirometer at a speed of 9 cm/s (200 rpm) for 20 min, the fish were challenged by swimming against the stream with increased water speed with an increment of 8.66 cm/s (100 rpm) (Ui), with each speed lasting for 2.5 min (Tii). When a fish became exhausted and unable to resume swimming from the downstream screen of the swim chamber, the maximum speed (Uii) and the passing time (Ti) within the cycle was recorded for this particular fish. After all fish were exhausted, the fish were allowed to recover for about 30 min and body length measurements (BL) were then determined. The critical swimming speed (Ucri) was calculated according to the following equation [47,48].

Ucrit=Ui+[Uii(Ti/Tii)]

### 2.11. Assessment of Fish Survival Curve And Ventricle Area

Fish deaths were recorded weekly starting from 3 months of age. The survival curve was drawn and the *p* value was calculated using JMP software. Ventricle area was measured in age matched and control and mutant fish group as described before [19].

### 2.12. Statistical Analysis

The values displayed in each graph are mean ± standard deviation. The non-parametric Wilson test was used to compare the mean from two different groups using JMP software (version 11, Cary, NC, USA). *p* < 0.05 was regarded as a significant difference between the two groups.

## 3. Results

### 3.1. Comparison of Gatad1 Gene Sequences between Zebrafish and Humans

**Figure 1:**
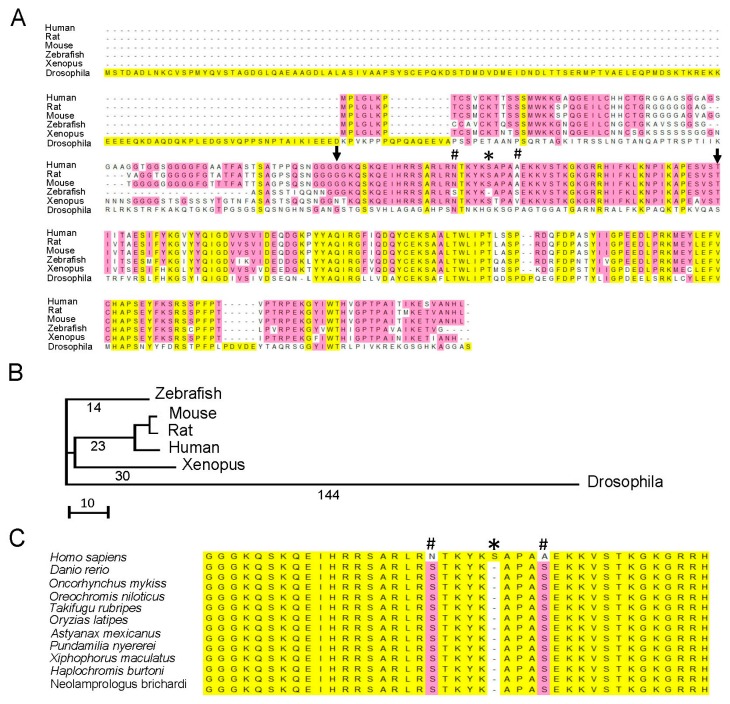
*Gatad1* is highly conserved between human and zebrafish. (**A**) Alignment of human GATAD1 protein sequence and five animal species. The human S102P mutation is localized to a region highly conserved in most animal models except Drosophila. Zebrafish does not have a serine residue in the position corresponding to human S102, but it has serine residues in positions corresponding to human N 97 and A106. # Human N 97 and A106; * Human S 102; arrows, range of highly conserved sequence from human G79 to I136; (**B**) The phylogenetic tree generated by comparing Gatad1 protein sequences in human and 5 animal species; (**C**) The alignment of peptide sequences flanking human S102 between human and fish species. All fish species have serine residue in the positions corresponding to human N 97 and A106, but not S102.

In humans, *GATAD1* is located on chromosome 7q21–7q22 and encodes a 269 amino acid protein. The corresponding zebrafish orthologue *gatad1* is located on chromosome 19 and encodes a 242 amino acid protein. There is a single transcript listed in the Ensembl database for zebrafish *gatad1* consisting of five exons (ENSDARG00000027612), which is similar to human *GATAD1* (ENSG00000157259). The genomic DNA sequence in the last four exons has 70%–82% sequence identity between zebrafish and human. The genomic DNA in exon 1 of zebrafish *gatad1* has 400 nucleotides, 360 of which have no significant similarity and 41 of which have 80% identity to corresponding sequence in exon 1 from the human *GATAD1* gene. The translation start site is localized to the 1st exon, while the peptide surrounding the human S102P mutation is encoded by the 2nd exon. At the protein level, there is 74% sequence identity between human GATAD1 and zebrafish Gatad1 proteins (Figure 1A). A *gatad1* orthologue can also be identified in the invertebrate *Drosophila*, but the sequence of the encoded protein is much more divergent from human GATAD1 (Figure 1B). Although there is remarkable conservation in the C-terminal half, the N-terminal half of the Gatad1 peptide including the domain surrounding the human S102P mutation is not conserved, rendering concern whether pathological changes resulting from the S102P mutation can be recapitulated in *Drosophila*. No *GATAD1* homologue was found in lower model organisms such as *Caenorhabditis elegans* and *Saccharomyces cerevisiae*.

In mammals, the peptide sequence surrounding the human GATAD1-S102P mutation, ranging from G79 to I136, is 100% conserved (Figure 1A). Interestingly, zebrafish Gatad1 is also highly conserved in this domain except for the residue corresponding to S102 in human GATAD1. In fact, zebrafish Gatad1 does not contain the serine residue in this position, but has an additional two serine residues in positions that correspond to N97 and A106 in human GATAD1. This unique sequence pattern is found in Gatad1 proteins from all fish species (Figure 1C), indicating an early evolutionary event that occurred when mammals started to diverge from fish.

### 3.2. Tissue Distribution of the Gatad1 mRNA

**Figure 2:**
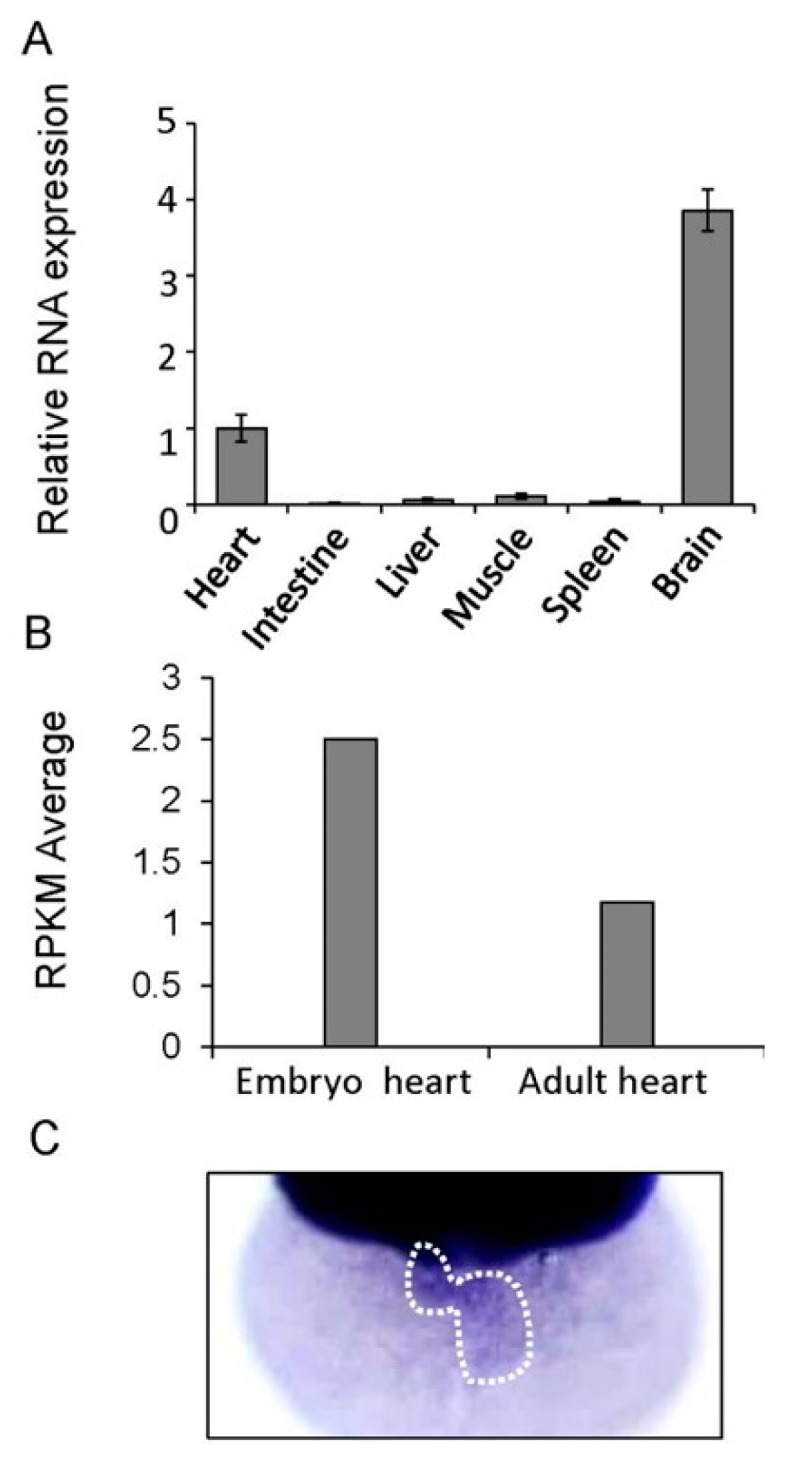
Assessment of the cardiac expression of *gatad1* in zebrafish. (**A**) The relative expression level of *gatad1* in different tissues were revealed by real-time RT-PCR using *18s* as internal control. Cardiac expression was defined as 1 and used as a reference. The brain has the highest *gatad1* expression; (**B**) Different expression levels of *gatad1* in embryonic and adult zebrafish hearts were revealed by RNA sequencing. *gatad1* expression in the embryonic heart is higher than that in the adult heart; (**C**) Cardiac expression of *gatad1* transcripts was also revealed by *in situ* hybridization in 2 dpf zebrafish embryos. RPKM: number of reads per kilobase per million reads.

We assessed *gatad1* mRNA expression in adult zebrafish tissues via real time RT-PCR. The relative expression of *gatad1* in heart is lower than in brain but higher than in intestine, liver, muscle and spleen (Figure 2A). We also examined absolute *gatad1* mRNA expression using our RNAseq data generated from both embryonic and adult zebrafish hearts [8]. We found that the expression of *gatad1* is 2.5 reads per kilobase per million reads (RPKM) in an embryonic fish heart and 1.1 RPKM in an adult fish heart. While our data indicated that although gatad1 does not have heart-enriched expression, it can be included with the 10,000 cardiac genes with RPKM > 0.3, *i.e.*, it has a meaningful absolute expression level in the heart (Figure 2B).

Whole-mount *in situ* hybridization (ISH) is a convenient technology in zebrafish embryos that can reveal tissue-specific expression patterns for a gene of interest. Cardiac expression of the *gatad1* transcript can be detected at 2 days post fertilization (dpf) after overstaining the embryos (Figure 2C). In addition to cardiac expression, we detected strong *gatad1* expression in the head, and weak expression in notochord, gut and pectoral fin (Supplementary Figure S1).

### 3.3. Subcellular Expression Pattern of Gatad1 Protein in the Nucleus and Sarcomeric I Band

**Figure 3:**
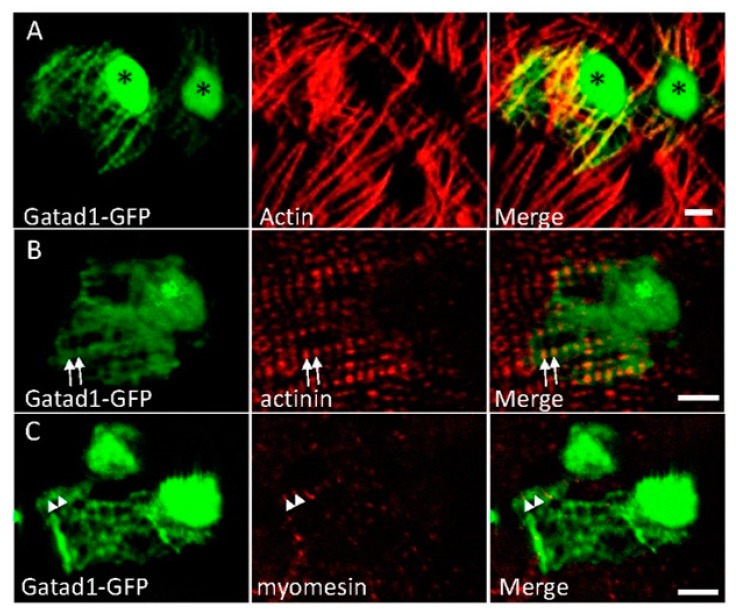
Subcellular localization of the Gatad1 protein in the embryonic zebrafish heart. After the *myl7:gatad1-GFP* construct was injected into 1-cell staged embryos, hearts from 2 dpf embryos were dissected for immunostaining and imaging. (**A**) Gatad1-GFP shows strong expression in nuclei and relatively weak expression in myofibrils, overlapping with Actin as revealed by phalloidin staining; (**B**) Gatad1-GFP partially overlaps with Z-discs marked by Actinin (arrows); (**C**) Gatad1-GFP forms alternatively striated patterns with M-line marked by Myomesin (arrowheads). * Nucleus; Scale bar 5 μm.

To determine the subcellular expression of the Gatad1 protein in the zebrafish heart, we generated a construct that encodes a GFP-tagged protein. After injecting the plasmid into a single cell stage zebrafish embryo, we isolated zebrafish hearts at 48 h post injection and determined the localization of GFP signal that is mosaically expressed. We noted strong GFP signals in the nuclei as well as the myofibril network, as revealed by co-staining Actin filaments with phalloidin (Figure 3A). Besides a diffuse expression pattern within cardiomyocytes, the Gatad1-GFP signal exhibited a striated pattern within the myofibril network. Double immunostaining studies demonstrated that the striated expression of Gatad1-GFP is attributable to co-localization with the I-band, as revealed by phalloidin, partial co-localization with the Z-disc, as revealed by an α-actinin antibody, but not co-localization with the M-line, as revealed by a myomesin antibody (Figure 3B,C). Together, these data indicate that Gatad1-GFP is localized to both the nucleus and the I-band region of myofibrils.

### 3.4. Heart Failure-Like Phenotypes in Gatad1 Knock-out Fish

**Figure 4:**
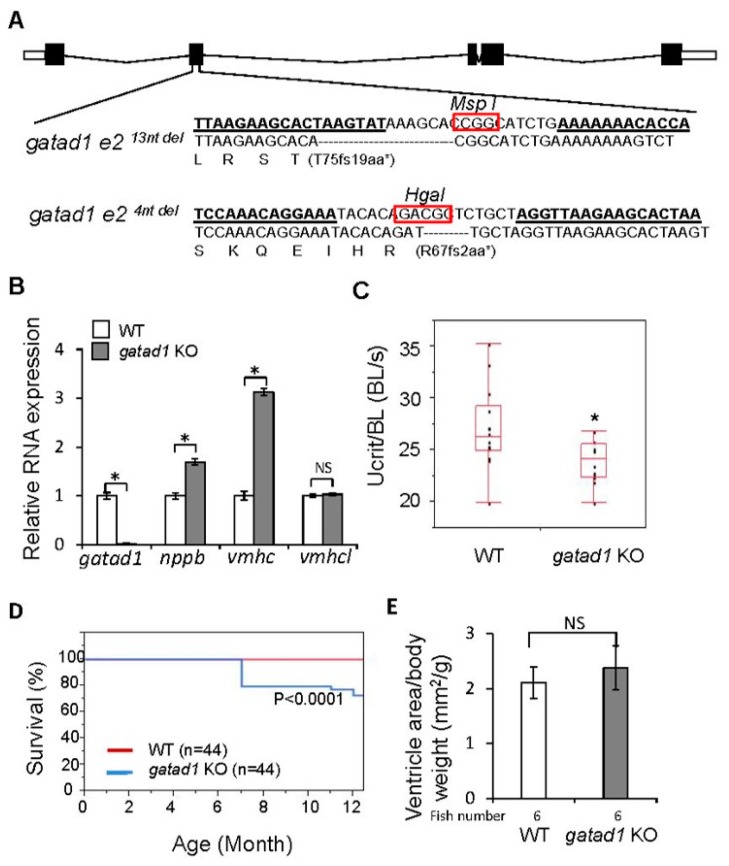
Generating and phenotyping *gatad1* knock-out zebrafish. (**A**) Two TALEN mutants for *gatad1* were generated. TALEN RNA binding sites within exon 2 are underlined. The restriction enzyme cut sites that facilitated genotyping are circled. The locations of the 13-bp and 4-bp deletions are shown below the wild type sequence, and the corresponding truncated peptides are indicated; (**B**) Fetal gene activation in the hearts of *gatad1* KO fish. The RNA expression levels of *gatad1*, *nppb*, *vmhc* and *vmhcl* are shown, as measured by real-time RT-PCR with *gapdh* as internal control. *gatad1* expression was reduced by 98% in *gatad1^13nt del^* homozygous mutant fish. The fetal genes *nppb* and *vmhc* were upregulated in *gatad1* mutant fish; (**C**) Significantly reduced swimming capacity was observed in *gatad1^13nt del^* homozygous mutant fish at 17 months. *gatad1^13nt del^* homozygous mutant fish and age-matched controls were stressed by 0.3% ethanol from 2 hpf to 48 hpf and then fed with 4% cholesterol from 2 months of age; (**D**) Significantly reduced survival rate was observed in *gatad1^13nt del^* homozygous mutant fish than in age-matched wild type fish after cholesterol and ethanol treatment; (**E**) Quantification of ventricle area shows no significant difference between wild type and *gatad1^13nt del^* homozygous mutant fish after cholesterol and ethanol treatment. * *p* < 0.05; WT, wild type; KO, knock-out. NS, not significant.

To further validate *GATAD1* as a cardiomyopathy gene, we generated *gatad1* homozygous mutants by designing a pair of TALEN RNAs targeting the 2nd exon of the zebrafish *gatad1* gene (Figure 4A). Two mutant alleles, *gatad1^4nt del^* and *gatad1^13nt del^*, were identified, which contained 4- and 13-base pair deletions that created different frameshifts. These small deletions would result in premature stop codons during translation, resulting in truncated Gatad1 proteins (Figure 4A). In fact, gatad1 RNA was reduced by 98% in fish heart isolated from homozygous *gatad1^13nt del^*, presumably due to nonsense-mediated mRNA decay (Figure 4B). Both *gatad1* knock-out mutant alleles survived to adult without any visually apparent phenotypes. The *gatad1^13nt del^* mutant was used for further experimentation. To determine if these fish were vulnerable to heart failure, we imposed stress by feeding a 4% cholesterol diet starting from 2 months of age. On this high cholesterol diet, *gatad1* homozygous mutant fish started to show reduced swimming capacity at 1.5 years of age (data not shown). To impose additional stress, *gatad1* mutant fish and age matched controls were treated with 0.3% ethanol during their embryonic stage from 2 hpf to 48 hpf, and then fed with 4% cholesterol starting from 2 months of age. Besides the reduced swimming capacity, we observed that *gatad1* homozygous mutant fish started to die after approximately 7 months of age and exhibited a significant reduction in survival rate comparing to age-matched wild type control fish (Figure 4C,D). Real-time RT-PCR revealed significant induction of *nppb* and *vmhc in gatad1* KO fish heart comparing to wild type fish heart. *Nppb* and *vmhc* are two fetal genes that have been used as molecular markers for hypertrophy [8], supporting a heart failure (HF) phenotypes (Figure 4B). Quantification of the heart size indicated a tendency for the group stressed with both ethanol and high cholesterol to show enlarged hearts (*p* = 0.2) (Figure 4E), but the group stressed with only a high cholesterol diet did not show enlarged hearts (data not shown).

### 3.5. Phenotypes in Transgenic Fish Line Expressing GATAD1 Containing the S102P Mutation

**Figure 5:**
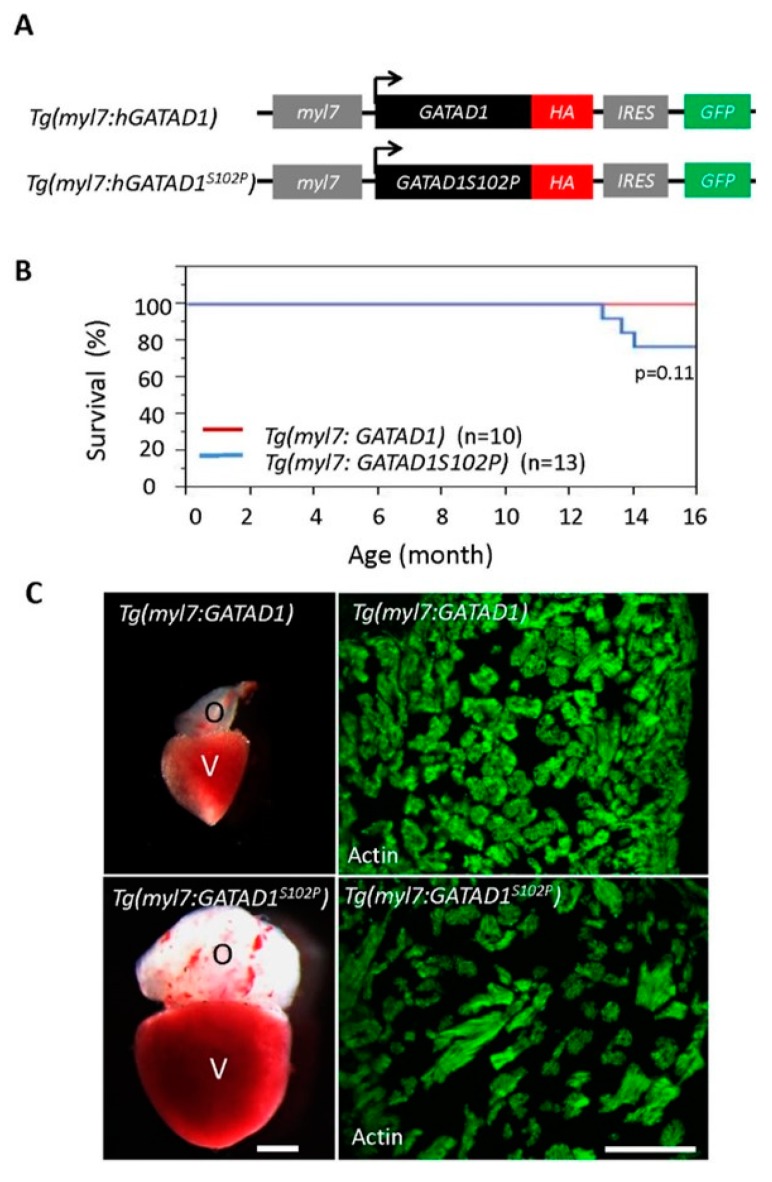
Generating and phenotyping *GATAD1* transgenic fish. (**A**) Schematic illustration of constructs that were used to generate two transgenic fish lines expressing either human wild type *GATAD1* or *GATAD1*-S102P mutation. The *GATAD1* gene is flanked by the myl7 enhancer at its 5′ terminal to drive cardiomyocyte-specific expression, and it has an HA tag and IRES-EGFP at its 3′ terminal to facilitate detection of ectopic gene expression and fish propagation; (**B**) The transgenic fish expressing mutant *GATAD1* started to die at approximately 12 months of age, and all transgenic fish expressing wild type *GATAD1* were able to survive to 16 months; (**C**) Severe cardiac hypertrophy in a *Tg(myl7:hGATAD1S102P)* fish. Left panel, images of a heart dissected from a *Tg(myl7:hGATAD)* and a *Tg(myl7:hGATAD1S102P)* fish at 17 months of age, separately. The heart from this single *Tg(myl7:hGATAD1S102P)* fish exhibits significantly enlarged ventricle and out flow tract. Right panel, phalloidin staining revealed less dense myofibril and wider myofibril in the heart of this single *Tg(myl7:hGATAD1S102P)* fish. V, ventricle; O, out flow tract; Scale bar 0.5 mm.

To model the GATAD1-S102P mutation identified in human DCM, we generated two stable transgenic fish lines. While *Tg(myl7:GATAD1)* expressed human wild type GATAD1 in cardiomyocytes, *Tg(myl7:GATAD1S102P)* expressed human GATAD1-S102P (Figure 5A). Expression of these ectopic genes was driven specifically in cardiomyocytes by a cardiomyocyte-specific *myl7* enhancer [49]. Both transgenic fish lines survived to the adult stage without visually abnormal phenotypes. However, the *Tg(myl7:GATAD1S102P)* fish started to die around 13 months of age, in contrast to *Tg(myl7:GATAD1)* fish that survived more than 20 months without any deaths (Figure 5B). There was no significant difference in swimming capacity between these two transgenic fish lines at 20 months of age (data not show), but 1 out of 6 *Tg(myl7:GATAD1S102P)* fish exhibited significantly increased heart size, increased width but decreased density in papillary muscles (Figure 5C). A larger sample size is required to validate this observation.

## 4. Discussion

### 4.1. Conservation of Zebrafish as a Model for Cardiomyopathy Genes

We used *GATAD1*, a recently identified gene for autosomal recessive DCM [36], as a paradigm to explore adult zebrafish as an *in vivo* model to validate cardiomyopathy gene mutations with age-dependent penetrance. Our data support the potential of adult zebrafish as an efficient model, but also reveal the limitations of this model that need to be addressed by further technology development.

At the protein level, zebrafish Gatad1 is conserved with human GATAD1. There is 70% amino acid identity, which is much higher than invertebrate models such as Drosophila. Importantly, over 50 amino acids flanking the human GATAD1 S102P mutation are almost completely identical between zebrafish and human. This high level of conservation justified our efforts to determine if zebrafish modeling would recapitulate the cardiomyopathy phenotype. Interestingly, zebrafish Gatad1 does not contain the serine residue in this position but has an additional two serine residues in positions that correspond to N97 and A106 in human GATAD1. It is possible that the two serine residues in fish species exert similar function as the single serine residue in mammalian GATAD1. Unfortunately, 3-D structure for GATAD1 has not been resolved yet, which prevented us from seeking structural evidence that would support this speculation.

### 4.2. Gene Expression Can be Profiled in Zebrafish

The expression profile of a gene usually provides important insights about its function and possibly its candidacy as a gene for heart disease. The zebrafish model offers the following three strategies to generate expression data for a candidate gene. First, *in situ* hybridization can be performed to reveal the three-dimensional gene expression profile during zebrafish embryogenesis. This is advantageous because zebrafish embryos develop *ex utero* and are available in large quantities. *In situ* data for more than one third of the genome have already been generated and deposited at Zfin (Zfin.org) [50]. Because the gene expression pattern of *gatad1* has not been reported in this database, we conducted our own *in situ* hybridization. We confirmed the cardiac expression of *gatad1*, and also revealed its expression in head, notochord, gut and pectoral fin. Second, we recently defined the zebrafish cardiac transcriptome at 4 dpf and 6 months using RNAseq technology, which can be used to determine the absolute cardiac expression level of a cardiomyopathy candidate gene [8]. Comparing to the expression of *gatad1* transcript in the embryonic heart, we found that the expression of *gatad1* in the adult heart is reduced by half. Third, tissue distribution of a candidate gene can be determined by real time RT-PCR using total RNA extracted from different organs. This is a technology that is also available in other animal models. *GATAD1* was also previously named *ocular development-associated gene* (*ODAG*), because the gene shows a dynamic expression pattern in the developing eye of mice [35]. Besides the eye, Gatad1 expression was detected in mouse ovary, testis, thymus, lung, kidney, spleen, liver, brain and heart [35]. Our real time RT-PCR data in fish are consistent with this multi-organ expression pattern, suggesting that GATAD1 may have other extra-cardiac functions.

The experimentally accessible zebrafish embryo provides an excellent platform for assessing the subcellular localization of proteins encoded by cardiomyopathy candidate genes. Localization can be studied by injecting a construct encoding a fluorescently tagged protein, making it unnecessary to develop an antibody. Imaging of an embryonic zebrafish heart can be facilitated by a heart isolation technique [42,51]. Of note, ISH can be conducted in sectioned adult zebrafish tissues [52,53], and sublocalization of a protein-of-interest can be followed in the adult stage using a stable transgenic fish line. In this study, we reported that zebrafish Gatad1 localizes to both the nucleus and I-band of the sarcomere. Similar cardiac expression patterns have been reported previously in humans by using a GATAD1 antibody [37]. The subcellular localization suggests future research directions to investigate Gatad1 as both a candidate transcription factor and sarcomeric protein, and uncover the pathogenic mechanism by which it leads to heart failure in DCM.

### 4.3. Genetic Engineering Tools are Available in Zebrafish for Modeling Cardiomyopathy

One of the most compelling pieces of evidence to validate a cardiomyopathy candidate gene is to recapitulate a cardiomyopathy phenotype in an animal model harboring a similar genetic mutation. At present, generating stable mutants for a candidate gene is no longer a bottleneck in the zebrafish model. Mutants for more than half of genes in the zebrafish genome have been generated by mutagenesis screening and distributed by Zfin. TILLING technology has also been used to produce mutants, and the lines have been distributed by the Zebrafish Mutagenesis Project at Sanger center [54]. Transposon-based mutagenesis screening has been used to produce mutants, and those mutants have been distributed by zFishbook and the International Protein trap consortium [55,56]. If mutants for a particular candidate gene are not available, targeted mutations for any gene can be easily generated by genome editing technology, as exemplified by the present study. If null mutants are needed, large deletions that completely remove the gene locus can be obtained by co-injecting two TALENs or CRISPR/Cas9 RNAs [57,58]. An important limitation of the loss-of-function (LOF) approach is evident in modeling human point mutations. If the missense mutation leads to pathogenesis through a gain-of-function (GOF) mechanism, then the LOF approach will not be able to recapitulate the disease pathogenesis. Knock-in technology would be needed to test these missense mutations, which is feasible in the zebrafish model. It has been shown that homologous recombination can be facilitated by co-injection of TALENs or Cas9 with either short oligos or long double-stranded DNA as a guide template [33,59,60].

As illustrated in the present study of *GATAD1*, many missense mutations cannot be mimicked by a simple knock-in approach to genetic manipulation. The missing serine residue at position 102 in fish species poses a significant challenge. It is plausible that two strategies can be used to address this challenge. In the first strategy, two steps of knock-in manipulation can be used. A zebrafish line containing a humanized Gatad1 can be initially generated by introducing a serine residue at position 102 and replacing the other two serine residues at positions 97 and 106 with N97 and A106, as in human GATAD1. A S102P mutation can be introduced into position 102 of this humanized fish line. A second strategy, as demonstrated in the present study, uses transgenic technology by directly expressing human GATAD1 with the S102P mutation in the zebrafish heart. Because of the efficient Tol2 transposon system, high transgenic efficiency can be achieved in zebrafish. The spatially-restricted expression of transgenes can be achieved by using tissue-specific enhancers, including the *titin* enhancer and the *myl7* enhancer for myocardium [49,51], the *fli* and *kdrl* enhancers for endocardium [61,62], and the *tcf 21* enhancer for epicardium [63]. The temporally-controlled expression of a transgene can be achieved by using the binary cre-loxP system [64]. There are certain limitations associated with transgenic models of cardiomyopathy, however, as have already been recognized in transgenic mouse models of HF in the past decade [65]. The ectopic gene expression level could be higher than a physiologically relevant level lead to non-specific cardiac remodeling process, as shown with GFP [66]. This concern can be partially addressed by including an appropriate control transgenic line, such as a line that expresses wild type human GATAD1.

We found that stable *gatad1* homozygous mutants did not exhibit an overt cardiac phenotype during embryogenesis or as adults under baseline conditions. This observation indicated that stable models are critical for assessing late-onset cardiomyopathies. The fact that phenotypes only show up in aged fish models is likely because of the late-onset nature of *GATAD1*-based cardiomyopathy. It is fully expected that early onset cardiomyopathy can be recapitulated in much younger adult zebrafish. Under imposed stress, we observed HF-like phenotypes in *gatad1* KO fish and a tendency to exhibit HF-like phenotypes in *GATAD1*-S102P transgenic fish that were not evident in the control transgenic line. The former observation suggested a haploinsufficient basis for GATAD1-based cardiomyopathy, which is consistent with the autosomal recessive segregation of DCM in the reported family it was discovered in [36]. On the other hand, the latter observation, if validated by future experimentation with larger sample size, suggests that the S102P mutation might also be able to exert a dominant negative effect in the heart. Further investigations in these genetic zebrafish models will clarify the mechanism of GATAD1-based DCM.

### 4.4. More Cardiomyopathy Phenotyping Tools are Needed in Adult Zebrafish

Because zebrafish is a new vertebrate model for cardiomyopathy, methodologies for defining cardiovascular pathophysiology in adult zebrafish are less developed than for rodent models. In this manuscript, we used several phenotyping tools to study the *gatad1* models. First, we assessed the survival of the mutant fish. In humans, survival is one of the most important indexes to assess heart failure. The 10-year mortality following diagnosis of dilated cardiomyopathy is approximately 40% [67]. The low cost of maintaining zebrafish colonies makes it possible to routinely assess survival of a HF model, even for a late-onset disease model such as *GATAD1*-based DCM. Previously, we demonstrated significantly reduced survival in acquired cardiomyopathy models induced by both anemia and DOX [21,24]. Here, we noted significantly reduced survival in *gatad1* KO fish and a tendency of reduced survival in S102P transgenic fish. Second, we used exercise intolerance as an index for HF in zebrafish. The treadmill testing is an important index for diagnosis of HF in rodent models [68]. Similarly, we detected reduced swimming capacity in *gatad1* KO fish, as would be expected with HF. It is anticipated that exercise intolerance will become an important non-invasive assay for future studies of HF in adult zebrafish. Third, to facilitate the detection of cardiomyopathy phenotypes, we imposed two stresses. Previous studies showed that feeding adult fish a high cholesterol diet induces hypercholesterolemia and atherosclerotic changes [44,45]. Additionally, ethanol exposure interrupts cardiac morphogenesis, causing heart defects in zebrafish embryos [46]. Maternal alcohol consumption can lead to fetal alcohol syndrome including heart defects in both human and animal models [69]. We reasoned that embryonic treatment with lower doses of ethanol would impose a more subtle impairment to the zebrafish heart, which might increase susceptibility to HF in the adult. Indeed, we found that stressing the fish with a high cholesterol diet can distinguish the *gatad1* KO from age-matched controls by showing reduced swimming capacity. When fish are stressed by both ethanol treatment and a high cholesterol diet, the *gatad1* KO fish exhibit both reduced swimming capacity and increased mortality.

Previously, we have established methodologies to define the hallmarks of cardiomyopathy in adult fish at both the cellular and molecular levels. At the cellular level, it is possible to quantify cardiomyocyte size by immunostaining with β-catenin or directly quantify isolated cardiomyocytes. Proliferation can be studied by immunostaining with PCNA. Apoptosis can be studied by the TUNNEL assay, and autophagy can be studied by Western blotting with LC3 [19,24]. At the molecular level, it is possible to assess re-activation of fetal genes such as *nppa*, *nppb*, and *vmhc* [8,24]. Here, we showed that both *nppb* and *vmhc* transcripts are upregulated in the *gatad1* KO fish hearts. Together with the increased mortality and reduced swimming capacity, our data support *GATAD1* as a causative gene for DCM.

In human patients, electrocardiography and echocardiography are two primary diagnostic tests for cardiomyopathy. Despite significant challenges imposed by the small size of the zebrafish heart (1~2 mm in diameter), both technologies are being developed and becoming feasible. Single lead electrocardiography has been developed to monitor heart conduction in anesthetized adult zebrafish [70,71]. A system that is capable of documenting zebrafish electrocardiograms is now commercially available from iWorx Inc. The technology was further advanced by the use of micro-electrodes [71,72]. Ultrasound technology to assess a zebrafish heart has also been actively developed over the past several years. Pulsed-wave Doppler signals can be readily measured to monitor blood flow in the heart, a surrogate of cardiac output [73,74]. However, measurement of ejection fraction to assess contractile function is relatively difficult to obtain because of limited resolution of the ultrasound machine and the difficulty in determining the border of a small zebrafish heart. 75-MHz-high-frequency ultrasound and Chirp-coded tissue harmonic imaging are being pursued to capture the real-time heart images and to characterize the zebrafish heart [73,75,76]. It is anticipated that further developments in non-invasive cardiac function technologies will further foster the use of adult zebrafish as an *in vivo* model for cardiomyopathy.

## 5. Conclusions

Our genetic studies of *gatad1* in adult zebrafish highlight the following unique features of this animal for studying cardiomyopathy. First, expression patterns of mRNA and protein at organ, cellular and subcellular levels can be efficiently determined. Secondly, stable gene knock-outs can be efficiently generated to model LOF mutants, and knock-ins and/or transgenes can be generated to study missense mutations that might exert GOF effects. Third, compared to mouse, maintaining a large number of fish colonies is relatively affordable, which is an important consideration when assessing diseases such as cardiomyopathy, which require extended longitudinal studies. Consequently, a single zebrafish lab can feasibly test dozens of candidate genes; such throughput is very difficult, if not impossible, with rodent models. At present, the small size of the zebrafish heart still poses significant challenges for detailed analysis of cardiovascular function. However, this bottleneck is being actively addressed by rapid technological advances. By complementing existing rodent and large mammal models of cardiomyopathy and heart failure, it is anticipated that the emerging adult zebrafish model will create new research opportunities and facilitate gene discovery, mechanistic understanding and therapeutic development for cardiomyopathy.

## Supplementary Materials

The following are available online at www.mdpi.com/2308-3425/3/1/6/s1, Figure S1: Tissue-specific expression of *gatad1* transcripts was revealed by in situ hybridization in 2 dpf zebrafish embryos.
