# Supplementary material for: Modeling GATAD1-Associated Dilated Cardiomyopathy in Adult Zebrafish
Source: J Cardiovasc Dev Dis. 2016 Jan 26;3(1):6. doi: 10.3390/jcdd3010006 (PMC5611887; doi:10.3390/jcdd3010006)
Supplement: Supplementary file 1 [file jcdd-03-00006-s001.pdf]

Supplementary Materials: Modeling *GATAD1*-Associated Dilated Cardiomyopathy in Adult Zebrafish

Jingchun Yang [1], Sahrish Shah [1], Timothy M. Olson [2,3] and Xiaolei Xu [1,2,*]

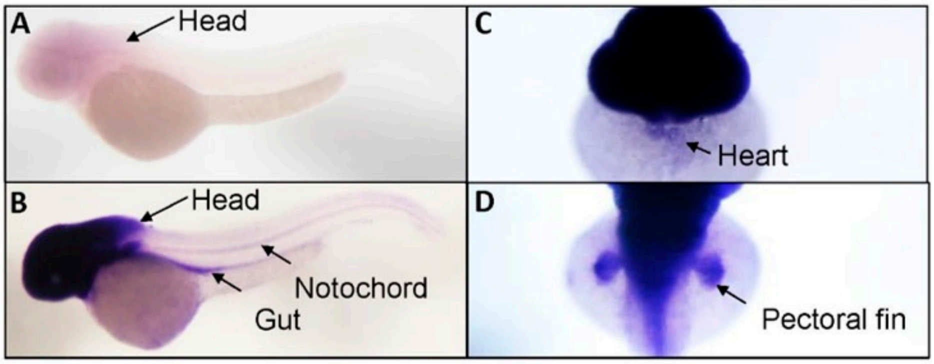

Figure S1. Tissue-specific expression of *gatad1* transcripts was revealed by *in situ* hybridization in 2 dpf zebrafish embryos. Short-time stain only revealed head expression of *gatad1* (**A**), but long time stain show *gatad1* expression in gut, notochord, heart and pectoral fin (**B**–**D**).
